# Cardiac Injury Biomarkers and the Risk of Death in Patients with COVID-19: A Systematic Review and Meta-Analysis

**DOI:** 10.1155/2021/9363569

**Published:** 2021-03-18

**Authors:** Sami H. Alzahrani, Mohammed W. Al-Rabia

**Affiliations:** ^1^Family Medicine Department, Faculty of Medicine, King Abdulaziz University, Jeddah, Saudi Arabia; ^2^Department of Medical Microbiology and Parasitology, Faculty of Medicine, King Abdulaziz University, Jeddah, Saudi Arabia

## Abstract

**Background:**

Cardiac complications may develop in a proportion of patients with the novel coronavirus disease (COVID-19), which may influence their prognosis.

**Objectives:**

To assess the role of cardiac injury biomarkers measured on admission and during hospitalization as risk factors for subsequent death in COVID-19 patients.

**Methods:**

A systematic review and meta-analysis was carried out involving cohort studies that compared the levels of cardiac injury biomarkers in surviving and dead COVID-19 patients. Cardiac injury is defined as an elevation of the definitive markers (cardiac troponin (cTnI and cTnT) and N-terminal pro-B-type natriuretic peptide (NT-proBNP)) above the 99th percentile upper reference limit. Secondary markers included creatine kinase-myocardial bound (CK-MB), myoglobin, interleukin-6 (IL-6), and C-reactive protein (CRP). The risk of death and the differences in marker concentrations were analyzed using risk ratios (RRs) and standardized mean differences (SMDs), respectively.

**Results:**

Nine studies met the inclusion criteria (1799 patients, 53.36% males, 20.62% with cardiac injury). The risk of death was significantly higher in patients with elevated cTn than those with normal biomarker levels (RR = 5.28, *P* < 0.0001). Compared to survivors, dead patients had higher levels of cTn (SMD = 2.15, *P*=0.001), IL-6 (SMD = 3.13, *P*=0.03), hs-CRP (SMD = 2.78, *P* < 0.0001), and CK-MB (SMD = 0.97, *P* < 0.0001) on admission and a significant rise of plasma cTnT during hospitalization.

**Conclusion:**

COVID-19 patients with elevated cTn on admission, possibly due to immune-mediated myocardial injury, are at increased risk for mortality. This requires further radiographic investigations, close monitoring, and aggressive care to reduce the risk of severe complications and death.

## 1. Introduction

The world has experienced an unprecedented pandemic of the novel severe acute respiratory syndrome coronavirus-2 (SARS-CoV-2) since the last days of 2019 when unexplained cases of lower respiratory tract infections have been reported in the Huanan Seafood Market, Wuhan, China [1]. The causative virus belongs to the *Coronaviridae* family, which comprises of a large group of coronaviruses (CoVs) that affect animals and humans. Similar to the highly identical virus SARS-CoV [2], the envelope spike protein (S protein) of SARS-CoV-2 binds to the angiotensin-converting enzyme 2 (ACE-2) receptors [3]. However, the novel virus has a 10- to 20-fold higher affinity for binding to its specific receptors than SARS-CoV [4], which may partly explain the widespread pattern among different populations. SARS-CoV-2 is transmitted from human to human, primarily via droplets and close contact [5]. The clinical spectrum of patient presentation ranges from the absence of apparent symptoms to severe respiratory failure [6]. Symptomatic patients usually present with fever, dry cough, dyspnea, myalgia, and fatigue; additionally, the majority of patients experience pneumonia.

In addition to the dominant respiratory symptoms, some COVID-19 patients might have severe cardiovascular damage. A clinical bulletin released by the American College of Cardiology [7] indicated the relevance of cardiac complications of the novel virus based on the fact that 12% of COVID-19 patients had had coassociated cardiac injury [6]. The risk of death might also be influenced by the history of a cardiovascular disease, and a proportion of patients may die as a result of cardiovascular deterioration along the course of the disease. Therefore, the role of cardiovascular biomarkers as predictors of future cardiac events might be significant in risk stratification on admission and during hospitalization. In particular, two isoforms of cardiac troponin (cTn), including cTnI and cTnT, have been widely used for the diagnosis of acute coronary syndrome [8]. In addition, natriuretic peptides, especially the N-terminal pro-B-type natriuretic peptide (NT-proBNP), are important biomarkers for diagnosis and prognosis of heart failure [9]. These laboratory markers have been reported in recent studies demonstrating the clinical characteristics of COVID-19 patients; however, few reports have emphasized the relevance of cardiac injury with the risk of death. The present systematic review and meta-analysis aimed to assess the association between the abnormal levels of cardiac injury biomarkers measured on admission and the subsequent risk of death among COVID-19 patients. Besides, we sought to explore the dynamic changes that have occurred during hospitalization in survived and deceased patients.

## 2. Methods

A systematic review and meta-analysis was conducted following the recommended guidelines of the Preferred Reporting Items for Systematic Reviews and Meta-Analyses (PRISMA) [10]. Eligible articles included retrospective and prospective cohort studies which reported the baseline laboratory findings of cardiac injury biomarkers in survived and deceased patients who had been diagnosed with COVID-19 according to the interim guidelines of the WHO [11] and had been confirmed using the reverse transcriptase polymerase chain reaction (RT-PCR) assay. Peer-reviewed and preprint articles were included. Definitive myocardial injury biomarkers included cTnI, cTnT, and NT-proBNP [12], and their elevated values in the serum (above the 99th percentile upper reference limit) have indicated cardiac injury. In addition to baseline values, dynamic laboratory findings of the definitive biomarkers during hospitalization in survived and nonsurvived groups were investigated. Moreover, baseline creatine kinase-myocardial bound (CK-MB), myoglobin, and the inflammatory parameters interleukin-6 (IL-6) and C-reactive protein (CRP) were considered as secondary biomarkers [12]. Studies recruiting specific populations of patients, such as critically ill and elderly, were excluded to optimize the generalizability of the results and to exclude the effects of aberrant cardiovascular biomarkers in such populations on the primary outcomes (mortality or survival). Review articles, case reports, and articles written in non-English language were ineligible.

Two independent authors, Dr. S.A. and M.A., performed the main search process in PubMed, Embase, and Google Scholar as of April 11, 2020. The date of publication was limited to 2020 when the outbreak emerged. Specific keywords were used in combination with Boolean operators (such as OR and AND); the strategy used in the PubMed database is provided in Appendix. The obtained records were exported to a specific program (Endnote X9, build 12,062) to organize references and delete duplicate records. The titles and abstracts of all the records were screened thoroughly, and the full-text versions of eligible studies were downloaded according to the availability of primary outcomes. Data extraction was performed in a spreadsheet (Microsoft Excel 2016) designed specifically for the study. Any disagreement between data collectors regarding study inclusion and data collection was resolved by discussion. The extracted data included (1) characteristics of the studies: the last name of the first author, country, setting, design, and period of data collection; (2) participants' data: the number of patients, gender, mean age of the total cohort, comorbidities at presentation, and the frequency of nonsurvival; (3) primary cardiac markers: baseline and in-hospital quantitative values of cTnI, cTnT, or NT-proBNP (pg/mL) and/or the frequency of patients with elevated biomarkers in serum; and (4) secondary biomarkers: baseline high-sensitivity CRP (hs-CRP) (mg/L), CK-MB (U/L), IL-6 (pg/mL), and myoglobin (ng/mL).

Numerical variables expressed as medians and interquartile ranges were converted to means and standard deviations (SDs) as indicated by Wan et al. [13]. The graphically represented data were converted to their respective numerical values after setting the accurate coordinates on images using GetData Graph Digitizer 2.26. The pooled case fatality rate and the prevalence of cardiac injury, as well as their respective 95% confidence intervals (95% CIs), were computed based on the weight of each study and the total number of patients. The standard error (SE) was calculated using the following formula: $\text{SE}=\sqrt{p\;\left(1-p\right)/n}$, where *p* indicates the prevalence and *n* indicates the sample size. All the quantitative and qualitative data were entered and analyzed in Review Manager software (version 5.3). For the analyses of the frequency of patients with elevated or normal cTn values (dichotomous variables), odds ratio and 95% CI expressed the difference in baseline comorbid conditions, whereas risk ratio (RR) and 95% CI were applied to assess the risk of death between both groups. Standardized mean differences (SMDs) and 95% CIs were used to analyze the difference in the quantitative values of cardiac biomarkers between survivors and nonsurvivors. The Mantel–Haenszel formula and inverse variance calculations were applied to the qualitative and quantitative analyses, respectively. The pooled effect estimates were computed using random-effects models if the heterogeneity between studies was significant (*I*^2^ > 50%); otherwise, a fixed-effects model was applied. Funnel plots were generated to explore publication bias.

The methodological quality was appraised by two authors (S.A. and M.A.) using the Strengthening the Reporting of Observational Studies in Epidemiology (STROBE) criteria for cohort studies [14], in which a score between 0 and 22 is given to each individual study based on a checklist of specific items related to the assessment of the title/abstract, introduction, methods, results, discussion, and other information. A STROBE score of <12 or 12–17 indicated a poor or moderate methodological quality, respectively.

## 3. Results

### 3.1. Results of the Search Process

**Figure 1:**
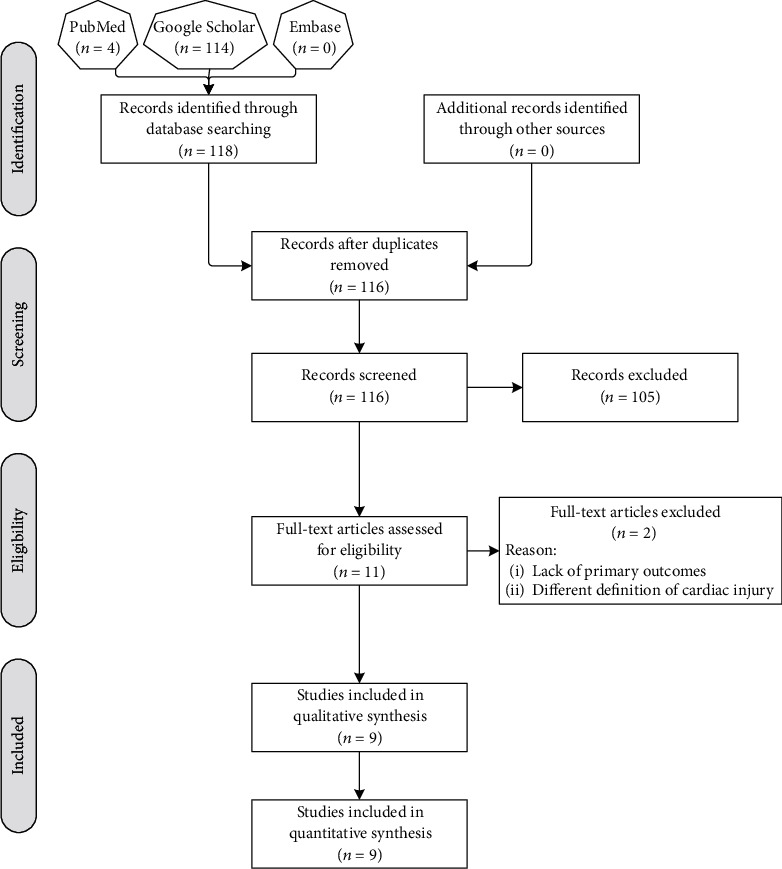
Flow chart showing the search process employed in the current study.

Figure 1 shows the results of the search strategy across different databases. Initially, 118 records were identified, of which two duplicates were omitted. The results of the screening process revealed a total of 11 studies meeting the eligibility criteria. However, after checking the full versions of eligible articles, two studies were excluded due to lack of primary outcomes in surviving and nonsurviving groups [15] and defining cardiac injury as elevated serum cardiac biomarkers above the 66th percentile upper reference limit [16]. Therefore, nine studies were formally included in the qualitative and quantitative analyses [17–25].

### 3.2. Characteristics of the Included Studies

The characteristics of the included studies are provided in Table 1. All studies were carried out in China, and they included retrospective analyses of medical records to collect patient data. Data collection was performed over a period between 21 and 52 days. A total of 1799 patients were included (53.36% males), and sample sizes ranged between 48 and 416 patients in a study. A history of hypertension was reported in 31.85% of patients, diabetes in 15.45%, coronary heart disease (CHD) in 9.41%, chronic obstructive pulmonary disease (COPD) in 3.88%, and malignancy in 3.41%. The methodological qualities of four studies [19, 21, 23, 25] were judged as “moderate,” whereas the remainder were of high methodological quality (Table 1). The overall weighted case fatality rate among hospitalized patients was 24.01% (95% CI 17.70–30.32, *I*^2^ = 91%, Supplementary Figure S1).

### 3.3. Levels of Cardiac Injury Biomarkers in Surviving and Deceased Patients

**Figure 2:**
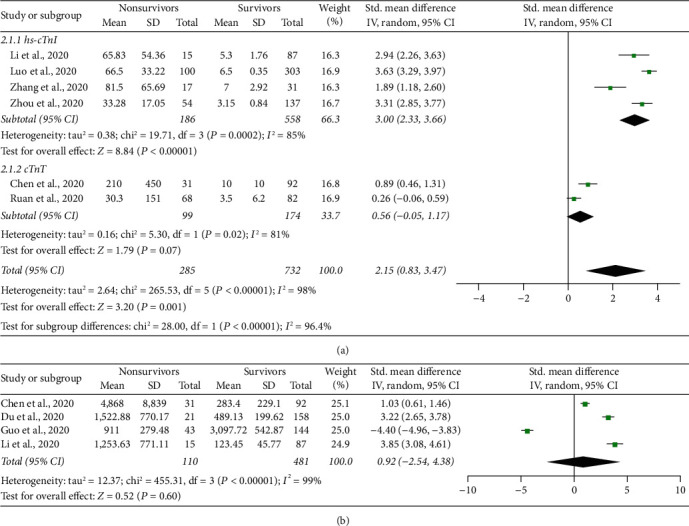
Forest plot showing the difference in the baseline mean values of cTn (a) and NT-proBNP (b) among surviving and nonsurviving COVID-19 patients.

Data regarding the mean cTn levels in survivors and nonsurvivors were available in six studies (1017 patients) [17, 19–21, 23, 24]. These included high-sensitivity cTnI (hs-cTnI) and cTnT. On admission, patients who did not survive had had significantly higher cTn levels compared to surviving patients (SMD = 2.15, 95% CI 0.83–3.47, *P*=0.001, *I*^2^ = 95%, *P*_*h*_ < 0.0001, Figure 2(a)). On subgroup analysis, the difference remained significant only for hs-cTnI (SMD = 3.00, 95% CI 2.33–3.66, *P* < 0.0001, *I*^2^ = 85%, *P* for heterogeneity (*P*_*h*_)=0.0002). There was no difference in the mean values of NT-proBNP between deceased and surviving patients (Figure 2(b)).

As for secondary cardiac and inflammatory biomarkers, compared to survivors, patients who did not survive had significantly higher levels of IL-6 (SMD = 3.13, 95% CI 0.24–6.03, *P*=0.03, *I*^2^ = 99%, *P*_*h*_ < 0.0001), hs-CRP (SMD = 2.78, 95% CI 0.90–4.65, *P*=0.004, *I*^2^ = 99%, *P*_*h*_ < 0.0001), and CK-MB (SMD = 0.97, 95% CI 0.62–1.32, *P*_*h*_ < 0.0001, *I*^2^ = 0%, *P*_*h*_=0.46, Table 2).

### 3.4. Baseline Parameters and Outcomes in Patients with Elevated or Normal cTn

Data regarding the cutoff values above which cTn was considered elevated were available in seven studies (1380 patients) [18–20, 22–25], whereas detailed clinical characteristics and outcomes in patients with and without cardiac injury were reported in two studies (603 patients) [18, 22]. The pooled prevalence of elevated cTn on admission was 20.62% (95% CI 17.22–24.03, *I*^2^ = 56%, Supplementary Figure S2). At baseline, patients with an initial cardiac injury had higher odds of preexisting CHD (OR = 8.49, *P* < 0.0001), COPD (OR = 6.50, *P*=0.0003), hypertension (OR = 5.43, *P* < 0.0001), and diabetes (OR = 2.98, *P* < 0.0001, Table 3). In addition, patients with elevated cTn had coelevated NT-proBNP (SMD = 5.59, 95% CI 3.00–8.17, *P* < 0.0001), CK-MB (SMD = 6.32, 95% CI 5.91–6.73, *P* < 0.0001), and hs-CRP (SMD = 4.69, 95% CI 3.82–5.55, *P* < 0.0001) compared to those with normal cTn values (Table 3).

**Figure 3:**
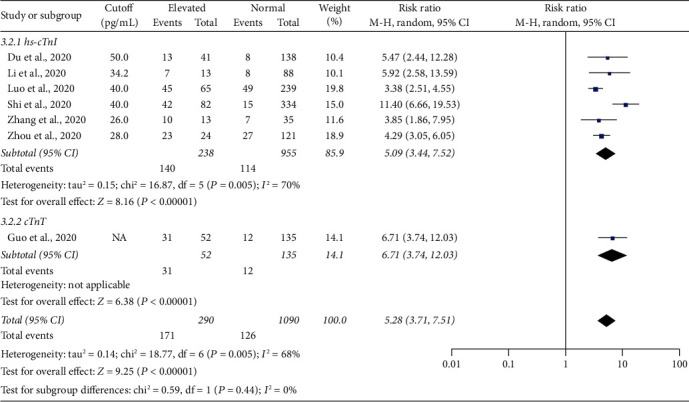
A forest plot showing the risk of death among COVID-19 patients with elevated and normal cTn on admission.

Regarding the outcomes, based on the reported data in seven articles [18–20, 22–25], the risk of death in patients with elevated cTn was significantly higher than that in their peers with normal biomarker values (RR = 5.28, 95% CI 3.71–7.51, *P* < 0.0001, *I*^2^ = 68%, *P*_*h*_ < 0.005, Figure 3); the risk was also higher when hs-cTnI or cTnT was measured (RR = 5.09 and 6.71, respectively, *P* < 0.0001 for both). The studies were distributed symmetrically around the pooled effect estimate, indicating no publication bias (Supplementary Figure S3).

### 3.5. Dynamic Changes during Hospitalization

The laboratory markers of COVID-19 patients were tracked during hospitalization in two studies [18, 24]. Zhou et al. [24] found that serum hs-cTnI levels increased in nonsurvivors (the median concentrations increased from 57.6 to 290.6 pg/mL during the period between day 16 and day 22 after the onset of infection, respectively), while they changed only slightly during hospitalization in recovered patients. However, trend changes were not analyzed statistically. In another retrospective analysis, Guo and coworkers [18] revealed statistically significant changes in the median values of plasma cTnT and NT-proBNP during hospitalization and before death compared to values reported on admission. Such temporal changes were not significant in cured patients.

## 4. Discussion

The cardiovascular implications of COVID-19 represent an important aspect of disease pathogenesis and prognosis. The present meta-analysis showed that about one in every five hospitalized patients had cTn levels above the 99th percentile upper reference limit, and elevated cTn on admission was associated with a fivefold increased risk of subsequent death compared to admitted patients with normal cTn (RR = 5.28). Moreover, absolute cTn levels became significantly elevated in patients who ultimately died due to COVID-19, and this was also associated with a baseline increase in CK-MB, IL-6, and hs-CRP. In addition to such baseline factors, the development of cardiac injury during hospitalization was evident among nonsurvivors.

Based on these findings, the effects of underlying cardiovascular comorbidities and myocardial damage on the prognosis for COVID-19 patients are significant. To exclude the impact of other confounding factors, multivariable adjusted models have shown independent associations between cardiac injury and mortality, with hazard ratios ranging between 4.26 and 10.90 [22, 23]. Furthermore, recent evidence showed that prior cardioprotective statin therapy has no significant effects on the subsequent COVID-19-related mortality [26]. Undoubtedly, cTn is a useful marker for cardiac injury in COVID-19 patients. The role of cTn measurement seems to be more critical in patients with decreased cardiovascular reserve, such as the elderly and those with preexisting cardiovascular comorbidities. Therefore, cTn monitoring, especially hs-cTn, is warranted in selected patients. Patients receiving antiviral drugs could also be monitored, since these medications can lead to arrhythmia, cardiac insufficiency, or cardiac toxicity [27, 28]. It appears that ordering cTn tests in COVID-19 patients should be based on a meticulous estimation of the pretest probability. Notwithstanding the increased sensitivity of hs-cTn assays, these tests come with a challenging reduction in the diagnostic specificity [29]. As such, the results should be interpreted in the context of preexisting conditions and the findings of electrocardiography and cardiac imaging. This is because false-positive cTn results caused by a low pretest probability would be associated with increased cardiology consults and extensive resource utilization [30].

Nonetheless, cardiac complications have been well established in outbreaks caused by coronaviruses. For example, it has been shown that severe acute respiratory syndrome (SARS) infections could be complicated by acute myocardial infarction, tachyarrhythmias, and signs of heart failure, although no preexisting cardiac diseases had been initially reported [31, 32]. Arrhythmias were also prevalent among 44% of patients in early reports following the emerged COVID-19 outbreak in Wuhan, China [33]. Atrial arrhythmias are the most commonly reported types, with atrial fibrillation consultations commonly requested during the peak of the COVID-19 outbreak in New York [34]. Other less common arrhythmias included ventricular arrhythmias, ventricular tachycardia storm, atrioventricular block, inappropriate sinus tachycardia, and postural orthostatic tachycardia syndrome [35–37].

Based on the aforementioned observations, it seems that there is a link between myocardial involvement and respiratory infections, and COVID-19 is no exception [38]. Severe hypoxemia resulting from acute respiratory failure contributes to reducing oxygen supply, which would stimulate the sympathetic system and increase myocardial oxygen demand [39, 40]. Indeed, postmortem studies have revealed that approximately 5–25% of patients who died from acute respiratory failure had experienced unobserved myocardial infarction [41]. Another possible mechanism is the proinflammatory state, where respiratory infectious agents can elicit an inflammatory pattern in atheromatous plaques [42]. Such a state of inflammation may mediate type I myocardial infarction via upregulating metalloproteinases and peptidases, destabilization of plaques, and thrombus formation [43]. Furthermore, inflammation may induce a prothrombotic state; thus, it promotes coronary thrombosis at sites of plaque detachment [44]. In COVID-19 patients, the pathophysiological changes associated with myocardial ischemia were apparent in the study of Shi et al. [22], where abnormal electrocardiogram (ECG) findings, such as ST-segment depression, T-wave depression and inversion, and Q waves, were reported in all the patients who had exhibited concomitantly elevated cardiac biomarkers.

Importantly, cardiac involvement in patients with COVID-19 may be related to micro- and macrothrombotic complications. A state of hypercoagulation has been reported elsewhere in the literature among patients with influenza [45, 46] and COVID-19 [47, 48]. Coagulopathy among COVID-19 patients is typically characterized by an elevated D-dimer concentration, prolonged prothrombin time, and a modest reduction in platelet count [49]. Multiple thrombogenic mechanisms have been proposed, such as stimulation of the complement cascade, RAS dysregulation, and thrombosis triggered by the cytokine storm [50]. Ackermann et al. [48] have shown a significantly higher prevalence of alveolar capillary microthrombi and pulmonary intussusceptive angiogenesis in autopsy specimens of COVID-19 patients compared to those with influenza. There is also growing evidence of the beneficial effects of in-hospital heparin on reducing COVID-related in-hospital mortality, whereas prehospitalization oral anticoagulation had had no effects on such a parameter [51]. This would further indicate the important role of microthrombosis in disease severity.

From another point of view, the mechanism of COVID-19-related myocardial injury could be explained by myocarditis, and this might be supported by the lack of acute respiratory distress in selected patients. Indeed, two main mechanisms may support such a hypothesis. First, direct viral spread from the respiratory tract through the circulatory or lymphatic systems may be responsible for these changes. The binding of SARS-CoV-2 to ACE-2 receptors, which are highly expressed in the heart [52], provides a rationale for such a hypothesis. Second, an imbalanced response of T cell populations can induce a rapid and massive production of cytokines, namely, a cytokine storm, causing an immunogenic myocardial injury. Subsequently, prominent cardiovascular changes may occur, such as increased vascular wall permeability, reduced coronary blood flow, coronary plaque destabilization, microthrombogenesis, and myocardial edema [6, 18]. Unfortunately, when focusing on studies providing detailed analyses of patients with elevated versus normal levels of cardiac injury biomarkers in the present meta-analysis [18, 22], cytokine-level measurement was unavailable, possibly due to logistical limitations.

Nevertheless, the concept of immune-mediated myocarditis could be supported by the fact that IL-6 was significantly associated with the pre- and postmanagement changes of cTnI levels in a recent case report of COVID-19 complicated with fulminant myocarditis [53]. Finally, as revealed in the current review, the coassociation of hs-CRP as a marker of inflammation with elevated cTn and the significant elevation of IL-6 in nonsurvivors may partly explain the immunogenic responses. The application of the monoclonal anti-IL-6 antibody tocilizumab in the management of COVID-19 pneumonia seems to be a promising solution [54, 55], and it is currently being considered in multiple clinical trials on patients who are more likely to develop multiple organ dysfunction and excessive cytokine release (ClinicalTrials.gov identifiers: NCT04332094, NCT04306705, NCT04345445, and NCT04339712).

There are some limitations that should be considered in the present meta-analysis. Patients' data were retrospectively collected, and thus, causal relationships between the risk of mortality and baseline parameters should be interpreted cautiously. Additionally, the included studies were all carried out in China, which might limit the generalizability of the results to other populations worldwide. From another perspective, the rationale of cTn testing was not reported in all studies, and the results of electrocardiography and echocardiography to confirm cardiac injury were not recorded. Data related to the clinical course of COVID-19 patients during the in-hospital stay were only available in two studies [18, 24], and thus, the quantitative synthesis of their outcomes was not possible. Importantly, the cause of death may be multifactorial, and it was difficult to emphasize the significance of myocardial injury as the sole and direct cause of death in each individual case. Finally, the small number of studies did not allow further investigation of the causes of heterogeneity via a quantitative analysis (Eggers' test) and the covariates that might have caused changes in the effect measures (meta-regression). These limitations would be resolved via conducting large-sized prospective studies that track the patterns of cTn requesting, monitor cardiac injury during hospitalization with cTn and radiographic techniques, assess the profiles of cytokines, and determine the causes of death, considering postmortem pathological changes.

## 5. Conclusion

Approximately one in five COVID-19 patients had a myocardial injury based on cTn testing on admission. Patients presenting with a cardiac injury had a fivefold increase in the risk of death compared to those with normal biomarkers. Deceased patients with a baseline cardiac injury had experienced an abnormal elevation of the inflammatory biomarkers, such as hs-CRP and IL-6, suggesting immune-mediated myocardial damage. Dead patients had also exhibited marked dynamic changes in cTn values during hospitalization, whereas survivors did not. Considering the implementation of high pretest probability, cardiac markers testing, particularly hs-cTnI, is advised on admission for selected patients, such as older adults and those with preexisting cardiovascular comorbidities. In addition, continuous monitoring during hospitalization is important to reduce the risk of cardiovascular complications and death.

## Figures and Tables

**Table 1:** Characteristics of the included studies.

Author	Design (country)	Settings	Period of data collection (days)	Sample size	Gender		Age	Survived	Deaths	STROBE score
					Male	Female				
Chen et al. [17]	RC (China)	Zhongnan Hospital and the Seventh Hospital of Wuhan city	45	123	61	62	57.79 ± 15.32	92	31	19
Du et al. [25]	RC (China)	Wuhan Pulmonary Hospital	43	179	97	82	57.60 ± 13.70	158	21	16
Guo et al. [18]	RC (China)	The Seventh Hospital, Wuhan	31	187	91	96	58.50 ± 14.66	144	43	20
Li et al. [19]	RC (China)	Tongji Hospital, Wuhan	35	102	59	43	57.25 ± 4.99	87	15	15
Luo et al. [20]	RC (China)	Eastern Campus of Renmin Hospital, Wuhan	27	403	193	210	54.75 ± 4.90	303	100	20
Ruan et al. [21]	RC (China)	Medical Records of NHC and China CDC	NA	150	102	48	56.82 ± 10.95	82	68	13
Shi et al. [22]	RC (China)	Renmin Hospital, Wuhan	21	416	205	211	61.00 ± 12.47	359	57	20
Zhang et al. [23]	RC (China)	Wuhan No. 1 Hospital	52	48	33	15	64.03 ± 16.54	31	17	17
Zhou et al. [24]	RC (China)	Jinyintan Hospital and Wuhan Pulmonary Hospital	33	191	119	72	56.25 ± 3.86	137	54	20

RC, retrospective cohort.

**Table 2:** Baseline differences in secondary cardiac injury biomarkers between COVID-19 survivors and nonsurvivors.

Biomarker	Studies	Patients			Heterogeneity		SMD (95% CI)	*P*
		Nonsurvivors	Survivors	Total	Model	*I* ^2^ (%)		
CK-MB	2	48	123	171	F	0	0.97 (0.62, 1.32)	<0.0001
hs-CRP	5	231	595	826	R	99	2.78 (0.90, 4.65)	0.004
IL-6	3	137	306	443	R	99	3.13 (0.24, 6.03)	0.03
Myoglobin	2	89	240	329	R	99	3.00 (−1.36, 7.36)	0.18

CI, confidence interval; CK-MB, creatine kinase-myocardial bound; CRP, C-reactive protein; F, fixed-effects model; IL-6, interleukin-6; R, random-effects model; SMD, standardized mean difference.

**Table 3:** Baseline differences between COVID-19 patients with elevated and normal cTn in terms of preexisting comorbidities and the mean values of other cardiac injury biomarkers.

Parameter	Category	Model	*I* ^2^ (%)	Effect estimate (95% CI)	*P*

Comorbidities	Hypertension	F	0	OR = 5.43 (3.60, 8.19)	<0.0001
	Diabetes	F	35	OR = 2.98 (1.84, 4.83)	<0.0001
	Coronary heart disease	F	44	OR = 8.49 (4.85, 14.86)	<0.0001
	COPD	F	23	OR = 6.50 (2.33, 18.09)	0.0003
	Malignancy	R	72	OR = 5.58 (0.90, 34.70)	0.07
	Chronic kidney disease	R	54	OR = 4.54 (0.80, 25.62)	0.09

Biomarkers	NT-proBNP (pg/mL)	R	98	SMD = 5.59 (3.00, 8.17)	<0.0001
	hs-CRP (mg/L)	R	84	SMD = 4.69 (3.82, 5.55)	<0.0001
	CK-MB (ng/mL)	F	0	SMD = 6.32 (5.91, 6.73)	<0.0001

^*∗*^Results are based on the analysis of 134 and 469 patients with elevated and normal cTn, respectively. CI, confidence interval; CK-MB, creatine kinase-myocardial bound; COPD, chronic obstructive pulmonary disease; F, fixed-effects model; hs-CRP, high-sensitivity C-reactive protein; OR, odds ratio; R, random-effects model; SMD, standardized mean difference.

## Data Availability

The data used to support the findings of this study are available from the Department of Family Medicine, King Abdulaziz University, Jeddah, Saudi Arabia.

## Appendix

### The Search Strategy Used in PubMed

  #1 “troponin” OR “high-sensitivity troponin” OR “TnI” OR “cTnI” OR “hs-cTnI” OR “hypersensitive cTn” OR “TnT” OR “cTnT” OR “hs-cTnT” OR “hypersensitive cTn”
  #2 “NT/proBNP” OR “NT-proBNP” OR “proBNP” OR “natriuretic peptide” OR “N-terminal pro-brain natriuretic peptide”
  #3 “creatinine kinase-myocardial band” OR “Creatine kinase MB” OR “creatinine kinase myocardial band” OR “CK-MB” OR “CKMB”
  #4 #1 OR #2 OR #3
  #5 “Survivor” OR “Survived” OR “Discharged”
  #6 “Non-survivor” OR “Deceased” OR “Death” OR “mortality” OR “died”
  #7 #5 AND #6
  #8 “coronavirus” OR “COVID-19” OR “SARS CoV-2”
  #9 #4 AND #7 AND #8

## Abbreviations

ACE-2: Angiotensin-converting enzyme 2
CHD: Coronary heart disease
CI: Confidence interval
CK-MB: Creatine kinase-myocardial bound
COPD: Chronic obstructive pulmonary disease
CRP: C-reactive protein
IL-6: Interleukin-6
MERS: Middle East respiratory syndrome
PRISMA: Preferred Reporting Items for Systematic Reviews and Meta-Analyses
RR: Risk ratio
RT-PCR: Reverse transcriptase polymerase chain reaction
SARS-CoV-2: Severe acute respiratory syndrome coronavirus-2
SD: Standard deviation
SMD: Standardized mean difference
STROBE: Strengthening the Reporting of Observational Studies in Epidemiology
WHO: World Health Organization.

## Supplementary Materials

Supplementary Materials Figure S1: the pooled case fatality rate among COVID-19 patients in the included studies. Figure S2: the pooled prevalence of elevated cTn among hospitalized patients with COVID-19. Figure S3: funnel plots investigating the publication bias for the outcomes of standardized mean differences in cTn (a) and NT-proBNP (b) and the risk of death among patients with normal or abnormal cTn (c).
