# Supplementary material for: Cardiac Injury Biomarkers and the Risk of Death in Patients with COVID-19: A Systematic Review and Meta-Analysis
Source: Cardiol Res Pract. 2021 Mar 18;2021:9363569. doi: 10.1155/2021/9363569 (PMC7977983; doi:10.1155/2021/9363569)
Supplement: Supplementary Materials — Figure S1: the pooled case fatality rate among COVID-19 patients in the included studies. Figure S2: the pooled prevalence of elevated cTn among hospitalized patients with COVID-19. Figure S3: funnel plots investigating the publication bias for the outcomes of standardized mean differences in cTn (a) and NT-proBNP (b) and the risk of death among patients with normal or abnormal cTn (c). [file 9363569.f1.docx]

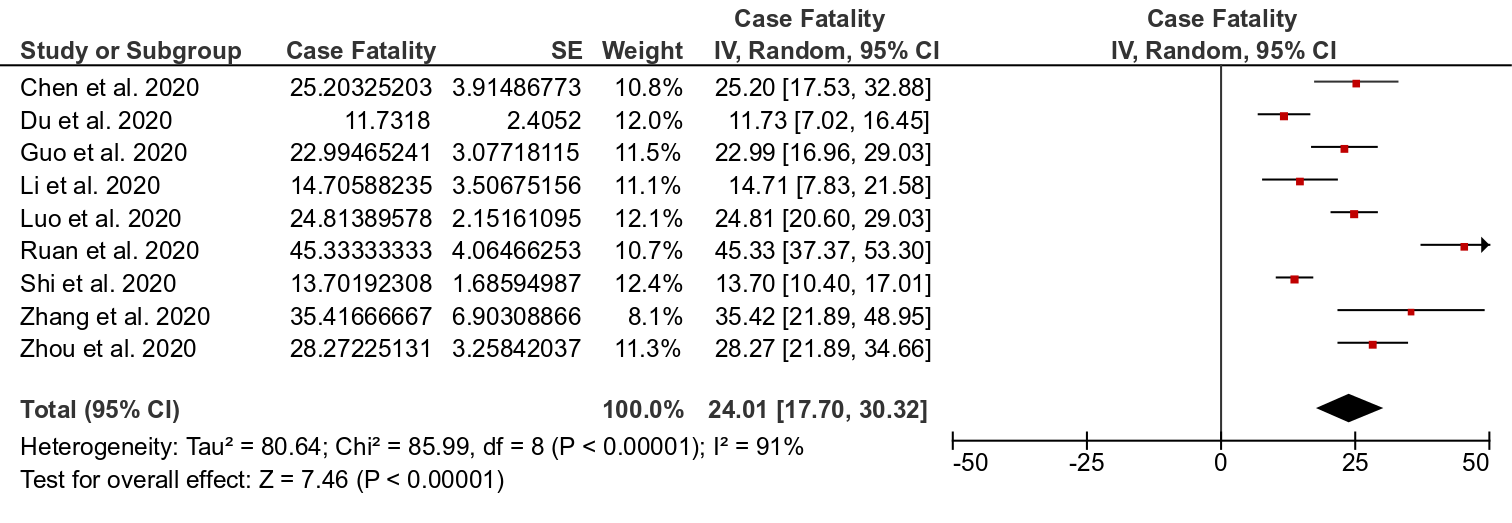


Figure S1: The pooled case fatality rate among COVID-19 patients in the included studies.


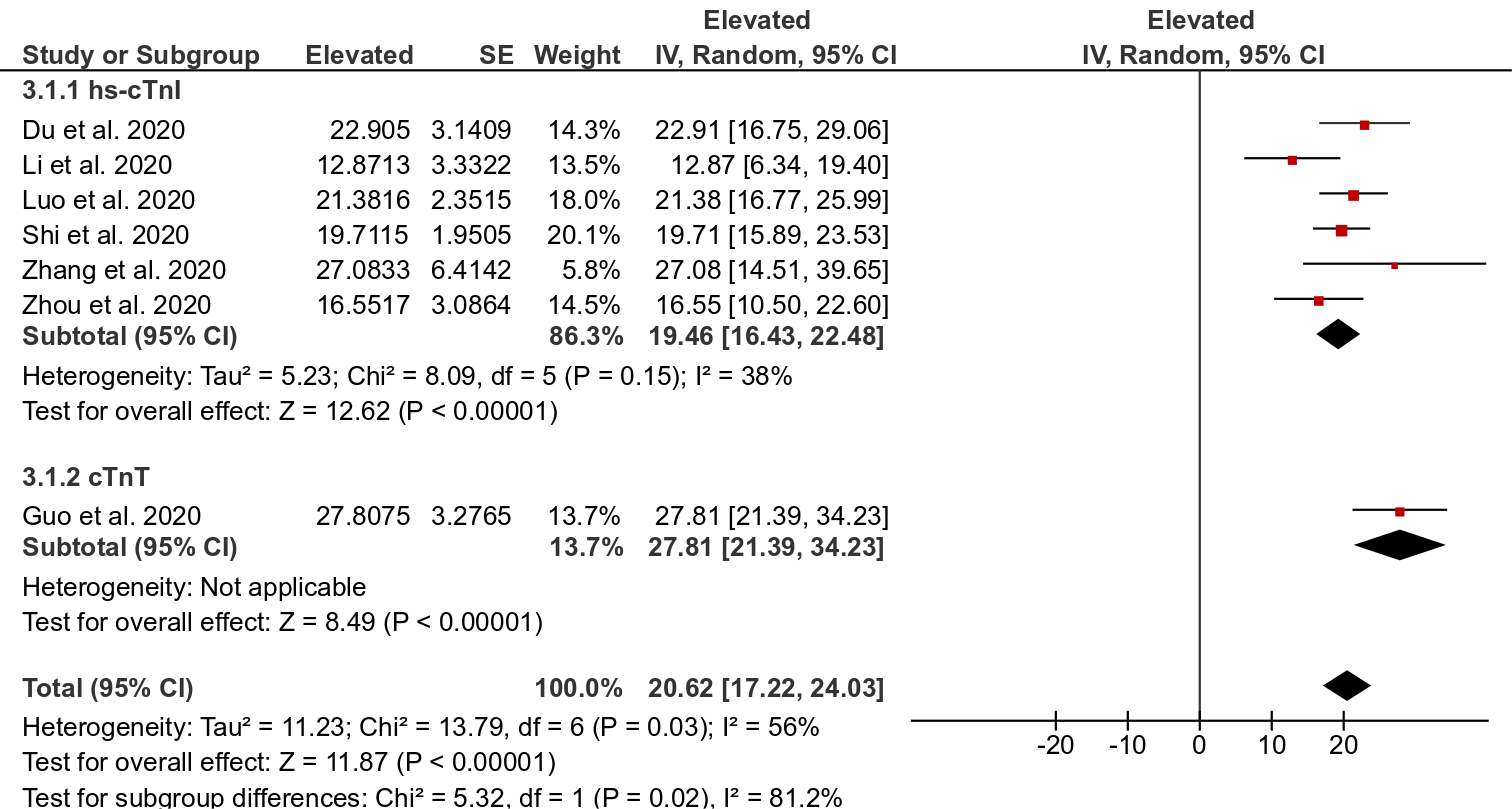


Figure S2: The pooled prevalence of elevated cTn among hospitalized patients with COVID-19.


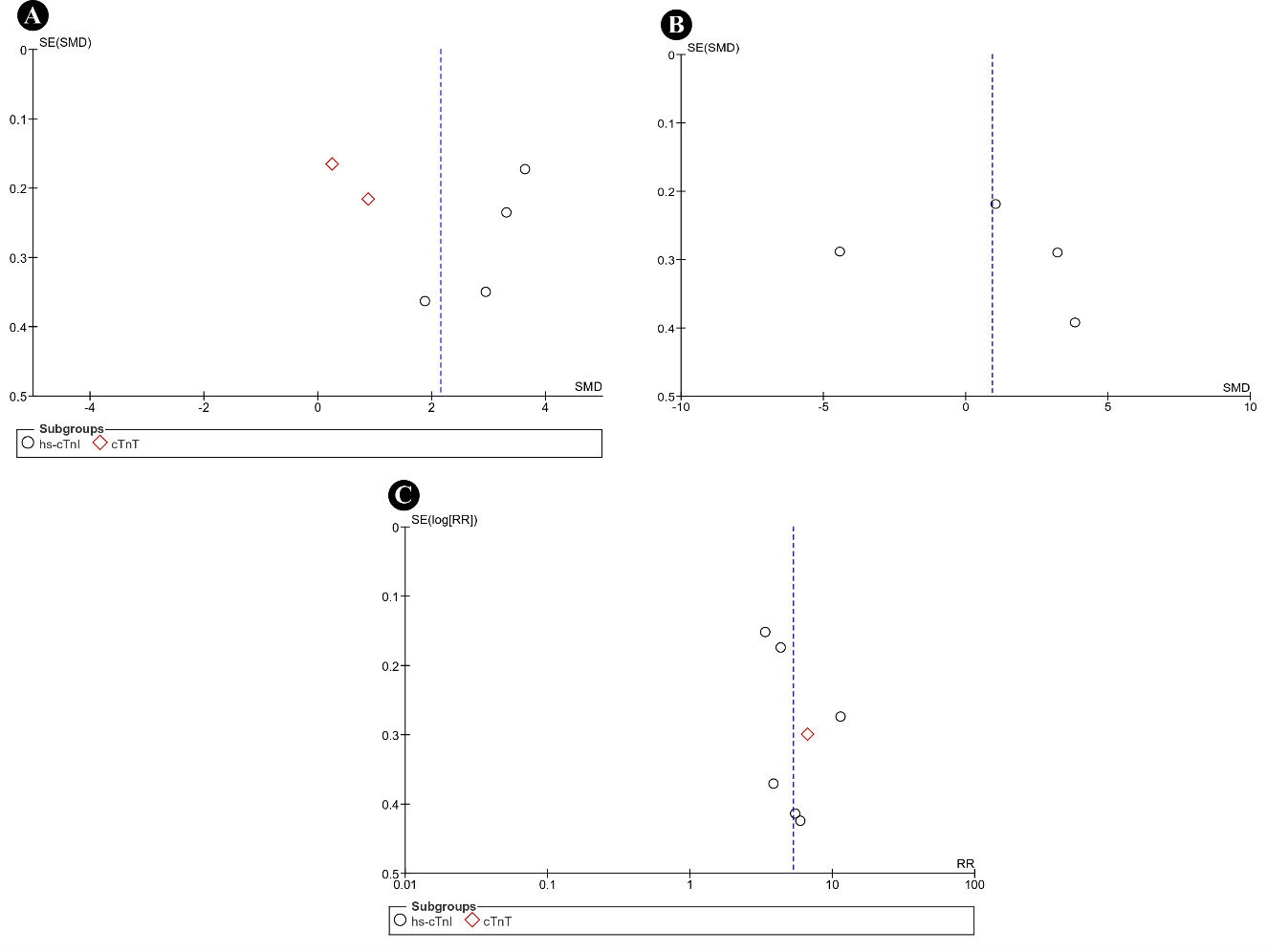


Figure S3: Funnel plots investigating the publication bias for the outcomes of standardized mean differences in cTn (A) and NT-proBNP (B) as well as the risk of death among patients with normal or abnormal cTn (C).
